# Supplementary figures and images for: Association of high-sensitivity C-reactive protein to albumin ratio with all-cause and cardiac death in coronary heart disease individuals: A retrospective NHANES study
Source: PLoS One. 2025 May 28;20(5):e0322281. doi: 10.1371/journal.pone.0322281 (PMC12119015; doi:10.1371/journal.pone.0322281)

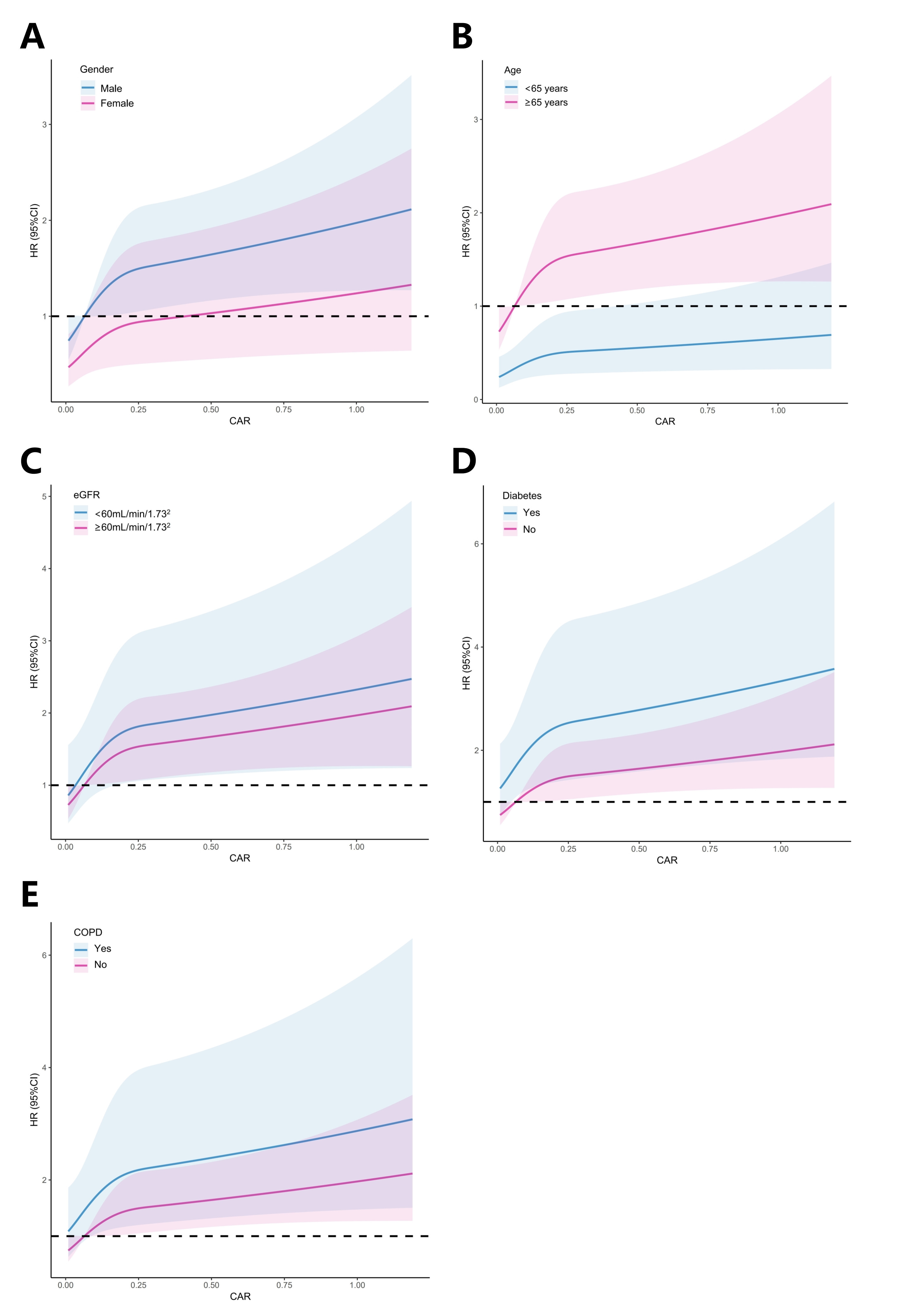

Supplement: S1 Fig — (JPG) [file pone.0322281.s001.jpg]
