# Supplementary material for: Association of high-sensitivity C-reactive protein to albumin ratio with all-cause and cardiac death in coronary heart disease individuals: A retrospective NHANES study
Source: PLoS One. 2025 May 28;20(5):e0322281. doi: 10.1371/journal.pone.0322281 (PMC12119015; doi:10.1371/journal.pone.0322281)
Supplement: S1 Table — (DOCX) [file pone.0322281.s002.docx]

**S1 Table.** Baseline characteristics of cardiovascular disease patients according to different hsCRP groups.

| **Variables** | **Total (624)** | **Low hsCRP (350)** | **High hsCRP (274)** | **P value** |
| --- | --- | --- | --- | --- |
| Age, years | 65.38 (12.03) | 67.28 (11.29) | 64.26 (12.16) | 0.001 |
| Female, n (%) | 257 (41.2) | 124 (35.4) | 133 (48.5) | 0.001 |
| Body mass index (kg/m^2^) | 30.24 (6.88) | 28.91 (5.75) | 31.94 (7.79) | < 0.001 |
| Physical exercise, n (%) | 298 (47.8) | 184 (52.6) | 114 (41.6) | 0.006 |
| Smoking, n (%) |  |  |  | 0.143 |
| Never | 311 (49.8) | 183 (52.3) | 128 (46.7) |  |
| Now | 95 (15.2) | 45 (12.9) | 50 (18.2) |  |
| Former | 218 (34.9) | 122 (34.9) | 96 (35.0) |  |
| Hypertension, n (%) | 133 (21.3) | 70 (20.0) | 63 (23.0) | 0.365 |
| Diabetes, n (%) | 234 (37.5) | 121 (34.6) | 113 (41.2) | 0.088 |
| Hyperlipidemia, n (%) | 516 (82.7) | 284 (81.1) | 232 (84.7) | 0.248 |
| Anemia, n (%) | 287 (46.0) | 136 (38.9) | 151 (55.1) | < 0.001 |
| COPD, n (%) | 156 (25.0) | 79 (22.6) | 77 (28.1) | 0.113 |
| Asthma, n (%) | 79 (12.7) | 37 (10.6) | 42 (15.3) | 0.076 |
| Depression, n (%) | 180 (28.8) | 77 (22.0) | 103 (37.6) | < 0.001 |
| Cancer, n (%) | 120 (24.2) | 65 (23.2) | 55 (25.6) | 0.542 |
| Segmented neutrophils (10^9^/L) | 4.51 (1.82) | 4.20 (1.64) | 4.91 (1.97) | < 0.001 |
| Platelet (10^9^/L) | 219.48 (62.88) | 208.78 (56.71) | 233.14 (67.65) | < 0.001 |
| Plasma glucose (mmol/L) | 6.40 (2.55) | 6.06 (1.77) | 6.82 (3.23) | < 0.001 |
| Total cholesterol (mmol/L) | 4.51 (1.14) | 4.41 (1.08) | 4.63 (1.20) | 0.016 |
| Triglycerides (mmol/L) | 1.74 (0.98) | 1.64 (0.91) | 1.87 (1.05) | 0.004 |
| LDL-C (mmol/L) | 2.48 (0.99) | 2.35 (0.86) | 2.65 (1.13) | 0.019 |
| HDL-C (mmol/L) | 1.29 (0.37) | 1.35 (0.39) | 1.21 (0.34) | < 0.001 |
| HsCRP (mg/L) | 5.36 (9.90) | 1.45 (0.77) | 10.35 (13.35) | < 0.001 |
| ALB (g/L) | 39.54 (3.56) | 40.51 (3.22) | 38.31 (3.59) | < 0.001 |
| AST (U/L) | 22.25 (10.01) | 22.73 (10.61) | 21.62 (9.17) | 0.168 |
| Creatinine (μmol/L) | 92.72 (46.78) | 89.89 (32.91) | 96.32 (59.85) | 0.111 |
| eGFR (mL/min/1.73m^2^) | 78.13 (28.71) | 79.03 (26.22) | 76.98 (31.61) | 0.389 |
| Outcomes |  |  |  |  |
| All-cause death | 97 (15.5) | 42 (12.0) | 55 (20.1) | 0.006 |
| Cardiac death | 35 (5.6) | 13 (3.7) | 22 (8.0) | 0.020 |
| Cancer death | 22 (3.5) | 10 (2.9) | 12 (4.4) | 0.306 |
