# Supplementary material for: Association of high-sensitivity C-reactive protein to albumin ratio with all-cause and cardiac death in coronary heart disease individuals: A retrospective NHANES study
Source: PLoS One. 2025 May 28;20(5):e0322281. doi: 10.1371/journal.pone.0322281 (PMC12119015; doi:10.1371/journal.pone.0322281)
Supplement: S2 Table — (DOCX) [file pone.0322281.s003.docx]

**S2 Table.** Baseline characteristics of cardiovascular disease patients according to different ALB groups.

| **Variables** | **Total (624)** | **Low ALB (300)** | **High ALB (324)** | **P value** |
| --- | --- | --- | --- | --- |
| Age, years | 65.96 (11.77) | 66.07 (11.35) | 65.85 (12.15) | 0.815 |
| Female, n (%) | 257 (41.2) | 146 (48.7) | 111 (34.3) | < 0.001 |
| Body mass index (kg/m^2^) | 30.24 (6.88) | 31.16 (7.57) | 29.40 (6.07) | 0.002 |
| Physical exercise, n (%) | 298 (47.8) | 126 (42.0) | 172 (53.1) | 0.006 |
| Smoking, n (%) |  |  |  | 0.731 |
| Never | 311 (49.8) | 146 (48.7) | 165 (50.9) |  |
| Now | 95 (15.2) | 49 (16.3) | 46 (14.2) |  |
| Former | 218 (34.9) | 105 (35.0) | 113 (34.9) |  |
| Hypertension, n (%) | 133 (21.3) | 60 (20.0) | 73 (22.5) | 0.441 |
| Diabetes, n (%) | 234 (37.5) | 127 (42.3) | 107 (33.0) | 0.016 |
| Hyperlipidemia, n (%) | 516 (82.7) | 233 (77.7) | 283 (87.3) | 0.001 |
| Anemia, n (%) | 287 (46.0) | 168 (56.0) | 119 (36.7) | < 0.001 |
| COPD, n (%) | 156 (25.0) | 82 (27.3) | 74 (22.8) | 0.195 |
| Asthma, n (%) | 79 (12.7) | 41 (13.7) | 38 (11.7) | 0.467 |
| Depression, n (%) | 180 (28.8) | 88 (29.3) | 92 (28.4) | 0.796 |
| Cancer, n (%) | 120 (24.2) | 57 (24.5) | 63 (24.0) | 0.914 |
| Segmented neutrophils (10^9^/L) | 4.51 (1.82) | 4.50 (1.88) | 4.52 (1.78) | 0.841 |
| Platelet (10^9^/L) | 219.48 (62.88) | 223.84 (69.17) | 215.4 (56.23) | 0.098 |
| Plasma glucose (mmol/L) | 6.40 (2.55) | 6.77 (3.12) | 6.05 (1.80) | 0.001 |
| Total cholesterol (mmol/L) | 4.51 (1.14) | 4.48 (1.18) | 4.54 (1.09) | 0.533 |
| Triglycerides (mmol/L) | 1.74 (0.98) | 1.66 (0.99) | 1.81 (0.98) | 0.060 |
| LDL-C (mmol/L) | 2.48 (0.99) | 2.47 (1.09) | 2.49 (0.90) | 0.884 |
| HDL-C (mmol/L) | 1.29 (0.37) | 1.30 (0.39) | 1.28 (0.36) | 0.580 |
| HsCRP (mg/L) | 5.36 (9.90) | 7.68 (12.75) | 3.21 (5.37) | < 0.001 |
| ALB (g/L) | 39.54 (3.56) | 36.75 (2.60) | 42.12 (2.05) | < 0.001 |
| AST (U/L) | 22.25 (10.01) | 22.14 (11.33) | 22.34 (8.63) | 0.809 |
| Creatinine (μmol/L) | 92.72 (46.78) | 98.62 (60.08) | 87.23 (28.51) | 0.003 |
| eGFR (mL/min/1.73m^2^) | 78.13 (28.71) | 75.58 (33.66) | 80.50 (23.00) | 0.035 |
| Outcomes |  |  |  |  |
| All-cause death | 97 (15.5) | 52 (17.3) | 45 (13.9) | 0.235 |
| Cardiac death | 35 (5.6) | 24 (8.0) | 11 (3.4) | 0.012 |
| Cancer death | 22 (3.5) | 10 (3.3) | 12 (3.7) | 0.802 |
