# Supplementary material for: Association of high-sensitivity C-reactive protein to albumin ratio with all-cause and cardiac death in coronary heart disease individuals: A retrospective NHANES study
Source: PLoS One. 2025 May 28;20(5):e0322281. doi: 10.1371/journal.pone.0322281 (PMC12119015; doi:10.1371/journal.pone.0322281)
Supplement: S3 Table — Multivariable analysis was adjusted for female age AST eGFR hypertension diabetes COPD and cancer. (DOCX) [file pone.0322281.s004.docx]

**S3** **Table.** Univariate and multivariable Cox regression analysis for predictors of all-cause death.

| **Variables** | **HR (95%CI)** | **P value** |
| --- | --- | --- |
| **Univariate analysis** | | |
| Age | 1.07 (1.05-1.10) | < 0.001 |
| Female | 0.56 (0.36-0.86) | 0.009 |
| Body mass index | 0.98 (0.95-1.01) | 0.174 |
| Physical exercise | 0.95 (0.64-1.41) | 0.794 |
| Diabetes | 2.03 (1.36-3.03) | 0.001 |
| Anemia | 0.94 (0.63-1.41) | 0.766 |
| Asthma | 1.64 (0.99-2.70) | 0.055 |
| Depression | 1.94 (1.29-2.90) | 0.001 |
| Segmented neutrophils | 1.21 (1.10-1.33) | < 0.001 |
| Platelet | 1.00 (0.99-1.00) | 0.092 |
| Plasma glucose | 1.00 (1.00-1.01) | 0.760 |
| Total cholesterol | 1.00 (0.99-1.00) | 0.090 |
| Triglycerides | 1.00 (1.00-1.01) | 0.815 |
| LDL-C | 0.76 (0.54-1.08) | 0.128 |
| HDL-C | 1.00 (0.98-1.01) | 0.579 |
| AST | 1.02 (1.10-1.04) | 0.046 |
| eGFR | 0.99 (0.98-0.99) | < 0.001 |
| Hypertension | 1.78 (1.16-2.73) | 0.009 |
| COPD | 1.84 (1.22-2.78) | 0.004 |
| Cancer | 2.46 (1.63-3.73) | < 0.001 |
| CAR | 1.51 (1.01-2.25) | 0.045 |
| hsCRP | 1.63 (1.09-2.44) | 0.018 |
| ALB | 0.80 (0.54-1.19) | 0.268 |
| **Multivariable analysis** | | |
| CAR | 1.77 (1.15-2.74) | 0.010 |
| hsCRP | 1.88 (1.22-2.90) | 0.004 |
| ALB | 0.71 (0.46-1.08) | 0.107 |

Multivariable analysis was adjusted for Age, Female, Body mass index, Diabetes, Asthma, Depression, Segmented neutrophils, Platelet, Total cholesterol, AST, eGFR, Hypertension, COPD and Cancer.
