# Supplementary material for: Association of high-sensitivity C-reactive protein to albumin ratio with all-cause and cardiac death in coronary heart disease individuals: A retrospective NHANES study
Source: PLoS One. 2025 May 28;20(5):e0322281. doi: 10.1371/journal.pone.0322281 (PMC12119015; doi:10.1371/journal.pone.0322281)
Supplement: S4 Table — (DOCX) [file pone.0322281.s005.docx]

**S4 Table.** Multivariable Cox regression analysis for predictors of all-cause and cardiac death at different follow-up times.

|  | **All-cause death** | | **Cardiac death** | |
| --- | --- | --- | --- | --- |
|  | **HR (95%CI)** | **P value** | **HR (95%CI)** | **P value** |
| Follow-up 3 year (n = 624) | | | | |
| CAR | 1.77 (1.15-2.74) | 0.010 | 2.99 (1.44-6.22) | 0.003 |
| hsCRP | 1.88 (1.22-2.90) | 0.004 | 2.63 (1.28-5.43) | 0.009 |
| ALB | 0.71 (0.46-1.08) | 0.107 | 0.41 (0.20-0.87) | 0.021 |
| Follow-up 5 year (n = 1076) | | | | |
| CAR | 1.72 (1.15-2.56) | 0.008 | 2.05 (1.10-3.79) | 0.023 |
| hsCRP | 1.81 (1.22-2.68) | 0.003 | 1.86 (1.01-3.43) | 0.046 |
| ALB | 0.43 (0.29-0.63) | < 0.001 | 0.31 (0.17-0.58) | < 0.001 |
