# Supplementary material for: Association of high-sensitivity C-reactive protein to albumin ratio with all-cause and cardiac death in coronary heart disease individuals: A retrospective NHANES study
Source: PLoS One. 2025 May 28;20(5):e0322281. doi: 10.1371/journal.pone.0322281 (PMC12119015; doi:10.1371/journal.pone.0322281)
Supplement: S1 File — (DOCX) [file pone.0322281.s006.DOCX]

# Supplementary Methods

Smoking^[1]^ status was classified as never smoking (<100 cigarettes in lifetime), former smoking (>100 cigarettes in life and smoke not at all now), and now smoking (>100 cigarettes in lifetime).

Hypertension was diagnosed by:

- SBP ≥ 140mmHg.
- DBP ≥ 90mmHg.
- Use of antihypertensive drugs.

Diabetes^[2]^ was diagnosed by:

- Self-reported doctor diagnosis of diabetes.
- Glycohemoglobin HbA1c > 6.5%.
- Fasting glucose ≥7.0 mmol/l.
- Random blood glucose ≥11.1 mmol/l.
- Use of diabetes medication or insulin.

Hyperlipidemia was diagnosed by:

- Hypertriglyceridemia: TG ≥ 150mg/dl.
- Hypercholesterolemia: TC ≥ 200mg/dl, LDL ≥ 130mg/dl.
- Low HDL: HDL < 40mg/dl (male),50mg/dl (female).
- Use of lipid-lowering drugs.

Anemia was diagnosed by:

- Male: hemoglobin ≤ 120g/L.
- Female: hemoglobin ≤ 110g/L.

COPD was diagnosed by:

- FEV1/FVC < 0.7 Post-Bronchodilator.
- Self-reported doctor diagnosis of emphysema.
- Subjects who are older than 40 years, have a history of chronic bronchitis, and use medications including selective phosphodiesterase-4 inhibitors, mast cell stabilizers, leukotriene modifiers and inhaled corticosteroids.

Asthma was diagnosed by:

- Self-reported doctor diagnosis of asthma.
- Use of antiasthmatic drug.

Depression was diagnosed and graded by the PHQ9 depression rating scale:

- Score 0-4: no depression.
- Score 5-9: mild depression.
- Score 10-14: moderate depression.
- Score ≥ 15: severe depression.

Cancer was diagnosed by:

- Self-reported doctor diagnosis of cancer.

eGFR was calculated by the formula:

$${186 \times(serum creatinine[mg/dL])}^{-1.154} \times{(age)}^{-0.203}\times(0.742 if female).$$

1. International Association for the Study of Lung Cancer. IASLC Language Guide. 2021, <https://www.iaslc.org/IASLCLanguageGuide.>
2. L. Stogdale. Definition of diabetes mellitus. *Cornell Vet*. 1986;76:156-174.
