# Supplementary material for: Association of high-sensitivity C-reactive protein to albumin ratio with all-cause and cardiac death in coronary heart disease individuals: A retrospective NHANES study
Source: PLoS One. 2025 May 28;20(5):e0322281. doi: 10.1371/journal.pone.0322281 (PMC12119015; doi:10.1371/journal.pone.0322281)
Supplement: Attachment — Submitted filename: Response to Reviewers.docx [file pone.0322281.s008.docx]

December 15, 2024

Dear Dr. Wu,

Thank you for your letter dated October 22, 2024, regarding our manuscript titled “Association of high-sensitivity C-reactive protein to albumin ratio with all-cause and cardiac death in coronary heart disease individuals: a retrospective NHANES study” (ID: PONE-D-24-23633). We sincerely thank the editors and reviewers for their valuable feedback, which helped improve the quality of our manuscript. We have revised the manuscript according to the editors’ and reviewers’ comments and have attempted to answer all their questions. In this letter, the comments are reproduced, and our responses are given just below them in a different color (red). We would also like to thank you for allowing us to resubmit our revised version of the manuscript. We hope that the revised manuscript is now suitable for publication in ***PLOS ONE***.

I look forward to hearing from you at your earliest convenience.

Sincerely,

Yaling Han MD, PhD

Cardiovascular Research Institute and Department of Cardiology,

General Hospital of Northern Theater Command Region, Shenyang 110016, China

Email: [hanyaling@163.net](mailto:hanyaling@163.net)

***Reviewer 1***

***Q1: Please provide a flowchart detailing the process of screening the population.***

**A1:** We are extremely grateful for your comments on the manuscript. The flowchart detailing the process of screening the population has been added to the manuscript as **Fig 1** in the **Materials and Methods Section**.

**Fig 1. The flowchart detailing the process of screening the population.**

***Q2: The authors categorized the CAR at 0.075 mg/g to stratify the levels of inflammation. Please demonstrate the optimal cutoff value from the ROC curve.***

**A2:** Thank you for the comments you put forward, highlighting the deficiency of the description in the manuscript. The ROC curve for cardiac death was calculated using logistic regression. The maximum value of the Youden index was 0.524, at which the CAR value was 0.0773486, the sensitivity was 0.743, and the specificity was 0.781 (**Table 1**). This was the optimal cut-off value. However, considering that the value of 0.077 is not convenient for calculation. Moreover, 0.075 mg/g was obtained from hsCRP = 3 (mg/L)/ALB = 40 (g/L). Therefore, it is undoubtedly more appropriate to use CAR = 0.075 mg/g.

**Table 1. The cut-off value of CAR calculated by the ROC curve .**

| **CAR value** | **Sensitivity** | **1-Specialty** | **Youden Index** |
| --- | --- | --- | --- |
| 0.0773486 | 0.743 | 0.219 | 0.524 |
| 0.0765809 | 0.743 | 0.221 | 0.522 |
| 0.0755362 | 0.743 | 0.222 | 0.521 |
| 0.0751677 | 0.743 | 0.224 | 0.519 |
| 0.0747171 | 0.743 | 0.226 | 0.517 |
| 0.074019 | 0.743 | 0.228 | 0.515 |
| 0.0735054 | 0.743 | 0.229 | 0.514 |
| 0.0729381 | 0.743 | 0.231 | 0.512 |
| 0.0667209 | 0.771 | 0.261 | 0.510 |
| 0.0725357 | 0.743 | 0.233 | 0.510 |
| ... | ... | ... | ... |

***Q3: The study results do not seem to support the descriptions in the discussion section. Please revise the discussion section carefully.For example, "In terms of cardiac death risk discrimination, CAR outperformed hsCRP and ALB, and the prediction model containing CAR had better predictive ability for all-cause death than ALB."***

**A3:** Thank you for your comment. For the sake of the concentration of the article content, we have deleted the relevant content of ROC curves, because this part makes a limited contribution to the main results of the manuscript. The statements in the **Discussion Section** have been modified.

“...In terms of cardiac death risk discrimination, CAR outperformed hsCRP and ALB...”

***Q4: There is an inconsistency between the text and the table, such as on line 185. Please correct this and check other parts for similar discrepancies..***

**A4:** Thank you for your comments. We carefully reviewed the results of the manuscript once again. When the subgroup analysis indicated that there was no interaction between CAR and each covariate, it was meaningless to analyze the differences in the death risk among the subgroups. Therefore, the part regarding the subgroup analysis of diabetic patients was deleted. The remaining table descriptions have also been meticulously cross-checked.

***Q5: The statements in lines 220 and 221 appear to be contradictory. Please clarify or resolve the inconsistency.***

**A5:** Thank you for your valuable comments. In some studies, CRP/ALB is called CAR, and in others, it is hsCRP/PAB. However, in our study, it is hsCRP/ALB. The cause lies in the fact that the patient information collected in each study varies, but the obtained conclusions are basically similar. To eliminate any ambiguity in expression, we have modified the **Discussion Section** of the manuscript.

“...that hsCRP/prealbumin (PAB) was an independent predictor of MACE using median dichotomization for hsCRP/PAB...”

***Q6: There are some spelling errors in the text. Please correct them accordingly.***

**A6:** Thank you for your meticulous examination. This error has already been rectified in the manuscript.

***Reviewer 2***

***Q1: The study aimed to explore the association of high-sensitivity C-reactive protein to albumin ratio with all-cause and cardiac death in coronary heart disease individuals by using the data from NHANES (2015-2018); however, I am curious about the delay in publishing these findings until 2024.***

**A1:** Thank you for your question. We equally express our regret for the problems you mentioned. The NHANES program suspended field operations in March 2020 due to the coronavirus disease 2019 (COVID-19) pandemic. As a result, data collection for the NHANES 2019-2020 cycle was not completed and the collected data are not nationally representative. Consequently, the data of the years 2019 - 2020 are beyond the consideration of this research.

Although the NHANES database was reactivated in August 2021, the NDI data associated with the NCHS was not entirely accessible. The follow-up data related to deaths was only updated until December 31, 2019. Thus, the data after 2021, although containing various covariates including CAR, lacked outcome events and were therefore excluded. Nevertheless, we anticipate that if there are more updates to the mortality data related to the NCHS, we will incorporate more studies with larger sample sizes to reinforce the conclusions of this research.

***Q2: It has been reported that geographic disparities in cardiovascular health are prevalent among American adults (doi: 10.1016/j.mayocp.2020.12.034). Did the authors consider this variability when selecting their samples?***

**A2:** Thank you very much for your valuable question. NHANES uses a stratified multistage sampling design to obtain a representative sample of US residents. The sampling plan consists of four stages: counties, segments, households and individuals. Moreover, the populations of different races have corresponding distributions in the groups. Therefore, the data we employed are representative to a certain extent. Nevertheless, given the small sample size of this study and the selection of the CHD population as the research subjects, a certain degree of bias may occur. The restriction of a slightly smaller sample size has been incorporated into the **Discussion Section**.

“...The sample size of the population studied was limited, and the diagnosis of CHD relied on a questionnaire survey rather than a large sample with accurately diagnosed individuals...”

***Q3: MR and CT imaging have emerged as promising complementary techniques for the primary diagnosis of coronary artery disease (CAD) and the detection of coronary atherosclerosis. There is publication based on the relationship between C-Reactive Protein/Albumin Ratio and Radiological Findings in Patients with COVID-19 (DOI:10.16899/jcm.900886). The authors might think to explore the inclusion of this relationship in their study.***

**A3:** Thank you for your comments and valuable suggestions. The article (***DOI:10.16899/jcm.900886***) aimed to determine the C-reactive protein/Albumin ratio (CAR) values of computed tomography (CT) -positive COVID-19 patients and CT-negative COVID-19 patients and to investigate the relationship between C-reactive protein/Albumin ratio and radiological images of patients. In cases where CT is contraindicated (such as pregnancy), CAR can be used to indicate lung involvement or to follow-up patients with pulmonary involvement.

However, there appears to be a disparity in the case of cardiovascular diseases. The imaging of cardiovascular diseases is the conventional examination for diagnosing CAD. Currently, an increasing number of studies are based on imaging examinations such as OCT, FFR and CT-FFR to make treatment decision choices. If the disease progresses to this stage, the value of imaging examinations is far superior to that of CAR. CAR can merely serve as a reference for determining the risk of adverse cardiovascular events with poor long-term prognosis in patients. Different from respiratory diseases, there are multiple diagnostic methods for CHD. Even if radiological examinations are not feasible, patients can still undergo non-invasive and non-radiation examinations like electrocardiography, echocardiography and hematological tests (such as cTNT and CK-MB) to determine the severity of CAD. Finally, although the risk of cardiac death in the CHD population with high CAR in this study was significantly higher than that in the low CAR group, there are considerable limitations in population selection. This study is only applicable to the community-based CHD population, and a portion was included based on the symptoms of angina. Due to variations in geographical and economic environments, it is unknown whether all or a suitable proportion of this population has the opportunity or is suitable for MRA/CTA examinations. Therefore, it is challenging to combine CAR and imaging examinations for risk prediction.

Nevertheless, an increasing number of institutions, including our research center, have initiated studies related to imaging in patients with CHD. Subsequently, the imaging indicators and CAR values of this in-hospital patient population can be directly utilized for research on the risk of adverse cardiovascular events. This will be an interesting research outcome and possess better clinical applicability. The relevant content has been discussed in the **Discussion Section** of the manuscript:

“...Finally, a portion of the population was included based on the symptoms of angina. Due to variations in disease severity, geographical and economic environments, it is unknown whether all or a suitable proportion of this population has the opportunity or is suitable for MRA/CTA examinations although they are conventional examinations for diagnosing CAD. Therefore, it is challenging to combine CAR and imaging examinations for risk prediction. However, with the increasing popularity of imaging examinations, it will be feasible to incorporate CAR and imaging examinations into risk stratification for prognosis-related research in the future....”

***Reviewer 3***

The main issue with the current study is the dispersion of its content and analyses. To examine the relationship between variables and outcomes, using limited but precise and detailed analyses is preferable to employing numerous unclear and incomplete approaches. The multitude of analyses and tables has resulted in many important points and explanations regarding the results being overlooked in both the tables and the text. Therefore, it is advisable to rewrite the manuscript while considering these points.

**A:** Thank you for your meticulous review and suggestions for this paper. After scrupulously contemplating the integrity of the article and the interpretability of the content, we re-wrote the manuscript and eliminated a portion of the content that was not highly relevant to the main results.

***Q1:*** Many of the variables mentioned in Table 1, which could be related to the study's outcomes, have been excluded in other analyses, leaving only a limited number included. What was the reason for this?

**A1:** Thank you for the question. Although some data, such as pulse, physical exercise and anemia, exhibited differences between the high CAR and low CAR groups in the baseline table, a univariate COX regression analysis revealed that they had no correlation with the primary outcome event of all-cause death. Hence, these indicators were excluded from the multivariate COX regression. However, in accordance with your suggestion in **Question 2**, all variables with a significance level lower than 0.2 in the univariate analysis were incorporated into the multivariate model to control confounding factors, maximize information and increase the statistical power of the study. The specific univariate and multivariate Cox regression tables are presented uniformly in **Question 2**, and the relevant statistical data in the manuscript has been rewritten.

***Q2: It is recommended that variables with a significance level below 0.2 or 0.18 in univariate analysis be included in the multivariate model (to control for confounders, maximize information, increase the statistical power of the study, etc.); however, this study only selected variables with a significance level below 0.05.***

**A2:** Thank you for the comments you put forward, highlighting the deficiency of the manuscript. In accordance with your suggestion, we have re-included all the variables with a significant difference of less than 0.2 in the univariate analysis into the multivariate Cox regression analysis and have rewritten the paper. Among them, there were an excessive number of missing values for LDL-C. Therefore, it was excluded from the multivariate Cox regression analysis. The results of the univariate and multivariate analyses related to all-cause and cardiac death are as follows:

**Table 1.** Univariate and multivariable Cox regression analysis for predictors of all-cause death.

| **Variables** | **Univariate analysis** | | **Multivariable analysis** | |
| --- | --- | --- | --- | --- |
|  | **HR (95%CI)** | **P value** | **HR (95%CI)** | **P value** |
| Age | 1.07 (1.05-1.10) | < 0.001 | 1.07 (1.04-1.10) | < 0.001 |
| Female | 0.56 (0.36-0.86) | 0.009 | 0.60 (0.37-0.98) | 0.043 |
| Body mass index | 0.98 (0.95-1.01) | 0.174 | 0.96 (0.92-0.99) | 0.034 |
| Physical exercise | 0.95 (0.64-1.41) | 0.794 | - | - |
| Diabetes | 2.03 (1.36-3.03) | 0.001 | 1.75 (1.73-2.71) | 0.012 |
| Anemia | 0.94 (0.63-1.41) | 0.766 | - | - |
| Asthma | 1.64 (0.99-2.70) | 0.055 | 1.18 (0.68-2.07) | 0.555 |
| Depression | 1.94 (1.29-2.90) | 0.001 | 2.19 (1.42-3.36) | < 0.001 |
| Segmented neutrophils | 1.21 (1.10-1.33) | < 0.001 | 1.13 (1.02-1.25) | 0.026 |
| Platelet | 1.00 (0.99-1.00) | 0.092 | 1.00 (1.00-1.01) | 0.830 |
| Plasma glucose | 1.00 (1.00-1.01) | 0.760 | - | - |
| Total cholesterol | 1.00 (0.99-1.00) | 0.090 | 1.00 (0.99-1.00) | 0.730 |
| Triglycerides | 1.00 (1.00-1.01) | 0.815 | - | - |
| LDL-C | 0.76 (0.54-1.08) | 0.128 | - | - |
| HDL-C | 1.00 (0.98-1.01) | 0.579 | - | - |
| AST | 1.02 (1.10-1.04) | 0.046 | 1.02 (1.00-1.04) | 0.048 |
| eGFR | 0.99 (0.98-0.99) | < 0.001 | 1.00 (0.99-1.01) | 0.656 |
| Hypertension | 1.78 (1.16-2.73) | 0.009 | 1.13 (0.72-1.80) | 0.592 |
| COPD | 1.84 (1.22-2.78) | 0.004 | 1.39 (0.89-2.20) | 0.152 |
| Cancer | 2.46 (1.63-3.73) | < 0.001 | 1.34 (0.86-2.08) | 0.201 |
| CAR | 1.51 (1.01-2.25) | 0.045 | 1.77 (1.15-2.74) | 0.010 |
| hsCRP | 1.63 (1.09-2.44) | 0.018 | 1.88 (1.22-2.90) | 0.004 |
| ALB | 0.80 (0.54-1.19) | 0.268 | 0.71 (0.46-1.08) | 0.107 |

Multivariable analysis was adjusted for Age, Female, Body mass index, Diabetes, Asthma, Depression, Segmented neutrophils, Platelet, Total cholesterol, AST, eGFR, Hypertension, COPD and Cancer.

Abbreviations: high-sensitivity C-reactive protein to albumin ratio, CAR; chronic obstructive pulmonary disease, COPD; low-density lipoprotein cholesterol, LDL-C; high-density lipoprotein cholesterol, HDL-C; high-sensitivity C-reactive protein, hsCRP; albumin, ALB; Aspartate Aminotransferase, AST; estimated glomerular filtration rate, eGFR.

**Table 2.** Univariate and multivariable Cox regression analysis for predictors of cardiac death.

| **Variables** | **Univariate analysis** | | **Multivariable analysis** | |
| --- | --- | --- | --- | --- |
|  | **HR (95%CI)** | **P value** | **HR (95%CI)** | **P value** |
| Age | 1.09 (1.04-1.13) | < 0.001 | 1.10 (1.04-1.15) | < 0.001 |
| Female | 0.35 (0.15-0.80) | 0.013 | 0.39 (0.17-0.94) | 0.036 |
| Body mass index | 0.98 (0.93-1.03) | 0.484 | - | - |
| Physical exercise | 0.95 (0.49-1.85) | 0.878 | - | - |
| Diabetes | 2.60 (1.32-5.11) | 0.006 | 1.90 (0.95-3.80) | 0.068 |
| Anemia | 0.90 (0.46-1.76) | 0.757 | - | - |
| Asthma | 1.13 (0.44-2.90) | 0.806 | - | - |
| Depression | 1.75 (0.89-3.44) | 0.106 | 2.01 (0.99-4.05) | 0.052 |
| Segmented neutrophils | 1.12 (0.94-1.32) | 0.201 | - | - |
| Platelet | 1.00 (1.00-1.01) | 0.652 | - | - |
| Plasma glucose | 1.00 (0.99-1.01) | 0.760 | - | - |
| Total cholesterol | 0.99 (0.98-1.00) | 0.077 | 1.00 (0.99-1.01) | 0.404 |
| Triglycerides | 1.00 (1.00-1.01) | 0.757 | - | - |
| LDL-C | 0.99 (0.98-1.01) | 0.263 | - | - |
| HDL-C | 0.99 (0.96-1.01) | 0.343 | - | - |
| AST | 1.02 (0.99-1.05) | 0.200 | 1.03 (1.00-1.06) | 0.096 |
| eGFR | 0.98 (0.97-1.00) | 0.007 | 1.00 (0.98-1.01) | 0.581 |
| Hypertension | 1.16 (0.53-2.55) | 0.715 | - | - |
| COPD | 2.02 (1.03-3.98) | 0.041 | 1.55 (0.78-3.09) | 0.214 |
| Cancer | 1.75 (0.84-3.65) | 0.133 | 0.93 (0.43-2.02) | 0.851 |
| CAR | 2.31 (1.15-4.65) | 0.019 | 2.99 (1.44-6.22) | 0.003 |
| hsCRP | 2.13 (1.07-4.23) | 0.031 | 2.63 (1.28-5.43) | 0.009 |
| ALB | 0.42 (0.21-0.86) | 0.018 | 0.41 (0.20-0.87) | 0.021 |

Multivariable analysis was adjusted for Female, Age, Diabetes, Depression, Total cholesterol, AST, eGFR, COPD and Cancer.

***Q3: For the classification of variables (such as smoking, diabetes, etc.), it is essential to provide references for the basis of classification.***

**A3:** Thank you for your suggestion. The relevant cited literature has been supplemented in the **Supplementary Methods**.

***Q4: The exclusion of individuals with missing hsCRP data may lead to selection bias, potentially skewing the results if those excluded had different mortality risks compared to those included.***

**A4:** Thank you for the comments. In the present study, there were a total of 1,189 participants suffering from CHD. Only 113 participants with missing CAR data were excluded, accounting for less than 10%. From a statistical perspective, this would not influence the accuracy of the results. Additionally, we made a statistical comparison of the mortality events between the 113 participants without CAR and the 624 participants who were ultimately included in the study, and there was no significant difference between the two.

**Table 3.** Outcomes of cardiovascular disease patients with or without CAR.

| **Variables** | **Total (624)** | **Total (113)** | **P value** |
| --- | --- | --- | --- |
| All-cause death, n (%) | 97 (15.5) | 22 (19.5) | 0.297 |
| Cardiac death, n (%) | 35 (5.6) | 6 (5.3) | 0.898 |
| Cancer death, n (%) | 22 (3.5) | 4 (3.5) | 0.994 |

***Q5: While 624 participants may seem adequate, it could be insufficient for subgroup analyses, particularly when examining interactions across multiple covariates. Small sample sizes in subgroups can lead to unreliable estimates.***

**A5:** Thank you for pointing out the shortcomings in this article. The study involving 624 participants was merely adequate for statistical analysis, but it is decidedly insufficient in real-world research, not to mention subgroup analysis. This limitation has been incorporated into the **Discussion Section**.

“...The sample size of the population studied was limited, and the diagnosis of CHD relied on a questionnaire survey rather than a large sample with accurately diagnosed individuals...”

***Q6: The U-shaped correlation between ALB and all-cause death risk is intriguing but requires further exploration and explanation. The implications of this finding for clinical practice are not sufficiently discussed.***

**A6:** We appreciate your indication of the shortcomings in this paper. The U-shaped risk relationship between ALB and all-cause mortality has been elaborated in detail in the **Results Section**.

“...In the U-shaped association between ALB and all-cause death risk, we obtained that the inflection point was 40.88, and subsequently calculated the effect size and confidence interval on the left and right sides of the inflection point by the segmented COX regression model. On the left side of the inflection point, each 1-unit increase in ALB was associated with a 22.6% reduction in the risk of unfavorable outcomes (HR: 0.85, 95%CI: 0.79-0.92, p < 0.001). On the right side of the inflection point, the effect size (HR) was 0.98 (95%CI: 0.85 to 1.15, p = 0.837)...”

The implications has benn expounded in the **Discussion Section**.

“...Furthermore, although a U-shaped correlation between ALB and the 3-year all-cause death risk in patients with CHD was observed, when the risk was calculated in segments using the inflection point of 40.88, it was found that on the left side of the inflection point, ALB was linearly and negatively correlated with the increase in all-cause death risk, while on the right side of the inflection point, the increase in ALB had no statistical significance in relation to the increase in death risk. Nevertheless, in clinical practice, while assessing the death risk of patients through CAR, we still need to pay attention to whether ALB is much higher than the normal value due to hemoconcentration or immune system diseases in patients, as this can cause bias in the judgment of outcome events...”

***Q7: Although Kaplan-Meier curves can be drawn based on cumulative hazard, since Kaplan-Meier and Cox models are fundamentally recognized as survival curves, it is better for the graphs to be drawn based on cumulative survival.***

**A7:** Thank you for your suggestion. Transforming the Kaplan-Meier curves into cumulative survival is more capable of accurately reflecting the difference in survival rates between patients in the high CAR and low CAR groups. We have already replaced the Kaplan-Meier curves in the **Result Section** of the manuscript.

***Q8: What was the purpose of using restricted cubic splines? There is no clear explanation regarding their results, and the discussion section does not tangibly address the dose-response relationship.***

**A8:** Thank you for the question you put forward. The objective of the RCS curve has been stated in the **Materials and Methods Section**.

“...Restricted cubic splines (RCS) were fitted to evaluate the dose-response relationship between CAR, hsCRP, ALB and the outcome events, and the nonlinearity was tested using likelihood ratio test with a threshold of P < 0.05...”

The results of the RCS curve have been explained in the **Results Section**.

“...Each one-unit increase in CAR is associated with a 78% increase in the risk of all-cause death (HR: 1.78, 95%CI: 1.17-2.69, p = 0.007) and a 164% increase in the risk of cardiac death (HR: 2.64, 95%CI: 1.45-4.80, p = 0.001). The trend in risk of death with increasing CAR and hsCRP was more pronounced in the lower ranges of CAR and hsCRP...”

“...In the U-shaped association between ALB and all-cause death risk, we obtained that the inflection point was 40.88, and subsequently calculated the effect size and confidence interval on the left and right sides of the inflection point by the segmented COX regression model. On the left side of the inflection point, each 1-unit increase in ALB was associated with a 22.6% reduction in the risk of unfavorable outcomes (HR: 0.85, 95%CI: 0.79-0.92, p < 0.001). On the right side of the inflection point, the effect size (HR) was 0.98 (95%CI: 0.85 to 1.15, p = 0.837)...”

And we discussed the meaning of RCS results in the **Discussion Section**.

“...Notwithstanding that the RCS curve has indicated that each unit increase in CAR is associated with a 78% increase in all-cause death risk and a 164% increase in cardiac death risk, it is not applicable for clinical analysis. Therefore, we used 3 mg/L divided by 40 g/L equals 0.075 mg/g as the cut-off value of CAR to distinguish between high inflammatory risk and low inflammatory risk...”
