# Supplementary material for: Association of high-sensitivity C-reactive protein to albumin ratio with all-cause and cardiac death in coronary heart disease individuals: A retrospective NHANES study
Source: PLoS One. 2025 May 28;20(5):e0322281. doi: 10.1371/journal.pone.0322281 (PMC12119015; doi:10.1371/journal.pone.0322281)
Supplement: Attachment — Submitted filename: Response_to_Reviewers_auresp_2.docx [file pone.0322281.s009.docx]

January 30, 2025

Dear Dr. Wu,

Thank you for your letter dated January 19, 2025, regarding our manuscript titled “Association of high-sensitivity C-reactive protein to albumin ratio with all-cause and cardiac death in coronary heart disease individuals: a retrospective NHANES study” (ID: PONE-D-24-23633). We sincerely thank the editors and reviewers for their valuable feedback, which helped improve the quality of our manuscript. We have revised the manuscript according to the editors’ and reviewers’ comments and have attempted to answer all their questions. In this letter, the comments are reproduced, and our responses are given just below them in a different color (red). We would also like to thank you for allowing us to resubmit our revised version of the manuscript. We hope that the revised manuscript is now suitable for publication in ***PLOS ONE***.

I look forward to hearing from you at your earliest convenience.

Sincerely,

Yaling Han MD, PhD

Cardiovascular Research Institute and Department of Cardiology,

General Hospital of Northern Theater Command Region, Shenyang 110016, China

Email: [hanyaling@163.net](mailto:hanyaling@163.net)

***Reviewer 4***

***Q1: Introduction***

***There are too many references grouped together, such as in lines 39 and 45 (e.g., [1-5], [9-13]). This can weaken the important studies that should be highlighted, and may include incorrect citations, for example, citation 14 refers to COVID-19, not CVD, and citation 16 is a review that is not very relevant to the statements made. The authors need to correct these errors and any similar issues.***

**A1:** Thank you for your suggestion. We have meticulously scrutinized each of the cited references to ensure their compliance with the citation standards. In **citation 14**, although CAR and other inflammatory indicators were mentioned, they were indeed irrelevant to CVD and have thus been deleted. Nevertheless, in **citation 16,** it was repeatedly stated that multiple studies have demonstrated that hsCRP is associated with the residual inflammatory risk of ASCVD and capable of guiding the treatment. The relevant statements are as follows, and we contend that they are pertinent to the description in this article.

The description in **citation 16**:

“...based on the magnitude of risk associations and availability of clinical assays, hsCRP emerged as the benchmark biomarker for inflammatory risk in ASCVD...”

“...A large body of evidence supports the use of hsCRP in primary prevention to guide therapeutic decision-making...”

“Although not yet part of routine clinical practice, hsCRP is also informative in secondary prevention. Most studies have observed positive associations between hsCRP and CV events...”

***Q2: Results***

***In the materials and methods section, two outcomes are mentioned: all-time cause of death and cardiac death (line 77). However, the results section also includes cancer death as an outcome. If cancer death is not part of all-cause death, the authors should define all-cause death and clarify the role of cancer in the overall analysis.***

**A2:** Thank you for the question. In the death events defined by NDI, the causes of death encompassed diseases of heart, malignant neoplasms, chronic lower respiratory diseases, accidents, cerebrovascular diseases, alzheimer’s disease, diabetes mellitus, influenza and pneumonia, nephropathy and other causes. Cancer death (malignant neoplasms) was a component of all-cause death. The definition of all-cause death was also appended to the **Materials and Methods section**, with the added content as follows. Cancer death was presented in the baseline data since cancer death was the second major cause of death in the study population after cardiac death, to observe whether there was any correlation between CAR and cancer death.

“...The causes of all-cause death encompasses diseases of heart, malignant neoplasms, chronic lower respiratory diseases, accidents, cerebrovascular diseases, alzheimer’s disease, diabetes mellitus, influenza and pneumonia, nephropathy and other causes...”

***Q3: Lines 222-224 contain an incomplete thought..***

**A3:** Thank you for your comments. The incomplete description has been rephrased as follows:

“...A Chinese community-based research involving 62,067 participants revealed that high levels of CAR can increase the risk of CVD. When screening high-risk populations for CVD, we should pay special attention to those with a simultaneous increase in CRP and a decrease in albumin...”

***Q4: Reference 23 is not relevant to the statement provided.***

**A4:** Thank you for the meticulous review. This citation is an incorrect citation and contains incomplete information, and has thereby been eliminated from the manuscript.

***Q5: Discussion***

***While the results are presented clearly, the discussion does not address the results effectively. This section could be enhanced by considering the potential effects of various covariates and the clinical and demographic characteristics of the study population on inflammation risk and CAR outcomes.***

**A5:** Thank you for the question, highlighting the deficiency of the manuscript. We have included the further elaboration of the results section within the **Discussion**, as follows:

“...Among the covariates, we found that gender, age, diabetes, depression, segmented neutrophils, AST, eGFR, hypertension, COPD and cancer were all significantly associated with all-cause death in univariate COX regression analysis. Moreover, the impact of diabetes, depression, hypertension, COPD and cancer on the risk of death was even greater than that of CAR. The reason is that all-cause death also includes deaths caused by these diseases, but not all of these diseases are related to inflammatory risk. Therefore, we believe that CAR is not accurate enough in predicting the risk of all-cause death. However, in terms of cardiac death, only gender, age, diabetes, eGFR and COPD were significantly associated with an increased risk of death in univariate COX analysis. In subgroup analysis, there was no significant difference in the association between CAR and the risk of death among subjects with different genders, ages, eGFR, history of diabetes and COPD. Moreover, the impact of these indicators on the risk of cardiac death was far lower than that of CAR, which not only demonstrated the correlation between cardiac death and inflammatory risk, but also ensured the more effective role of CAR in predicting the risk of cardiac death in CHD population...”

***Q6: References***

***Authors need to ensure that references are cited correctly***

**A6:** Thank you for your suggestion. Each reference citation has been meticulously examined to ensure its compliance with the citation requirements and its correct formatting.
